# Driver mutations (*JAK2*V617F*, MPL*W515L/K or *CALR*), pentraxin-3 and C-reactive protein in essential thrombocythemia and polycythemia vera

**DOI:** 10.1186/s13045-017-0425-z

**Published:** 2017-02-22

**Authors:** Federico Lussana, Alessandra Carobbio, Silvia Salmoiraghi, Paola Guglielmelli, Alessandro Maria Vannucchi, Barbara Bottazzi, Roberto Leone, Alberto Mantovani, Tiziano Barbui, Alessandro Rambaldi

**Affiliations:** 1 Hematology and Bone Marrow Transplant Unit, ASST Papa Giovanni XXIII Bergamo, Piazza OMS, 1, 24127 Bergamo, Italy; 2 Research Foundation, ASST Papa Giovanni XXIII, Bergamo, Italy; 3 Department of Experimental and Clinical Medicine, CRIMM, Center Research and Innovation of Myeloproliferative Neoplasms, Azienda Ospedaliera Universitaria Careggi, University of Florence, Florence, Italy; 4 Humanitas Clinical and Research Center, Rozzano, Milan, Italy; 5 Humanitas University, Rozzano, Milan, Italy; 6 Department of Oncology and Hematology, University of Milan, Milan, Italy

**Keywords:** Polycythemia vera, Essential thrombocythemia, Mutations, Inflammation

## Abstract

**Background:**

The driver mutations *JAK2*V617F, *MPL*W515L/K and *CALR* influence disease phenotype of myeloproliferative neoplasms (MPNs) and might sustain a condition of chronic inflammation. Pentraxin 3 (PTX3) and high-sensitivity C-reactive protein (hs-CRP) are inflammatory biomarkers potentially useful for refining prognostic classification of MPNs.

**Methods:**

We evaluated 305 with essential thrombocythemia (ET) and 172 polycythemia vera (PV) patients diagnosed according to the 2016 WHO criteria and with full molecular characterization for driver mutations.

**Results:**

PTX3 levels were significantly increased in carriers of homozygous *JAK2*V617F mutation compared to all the other genotypes and triple negative ET patients, while hs-CRP levels were independent of the mutational profile. The risk of haematological evolution and death from any cause was about 2- and 1.5-fold increased in individuals with high PTX-3 levels, while the thrombosis rate tended to be lower. High hs-CRP levels were associated with risk of haematological evolution, death and also major thrombosis. After sequential adjustment for potential confounders (age, gender, diagnosis and treatments) and the presence of *JAK2*V617F homozygous status, high hs-CRP levels remained significant for all outcomes, while *JAK2*V617F homozygous status as well as treatments were the factors independently accounting for adverse outcomes among patients with high PTX3 levels.

**Conclusions:**

These results provide evidence that *JAK2*V617F mutation influences MPN-associated inflammation with a strong correlation between allele burden and PTX3 levels. Plasma levels of hs-CRP and PTX3 might be of prognostic value for patients with ET and PV, but their validation in future prospective studies is needed.

## Background

Polycythemia vera (PV) and essential thrombocythemia (ET) are chronic myeloproliferative neoplasms (MPNs) characterized by clonal expansion of an abnormal haematopoietic progenitor cell and a clinical course that is complicated by frequent cardiovascular complications as well as increased risk of transformation to myelofibrosis (MF) or acute leukaemia (AL) [1, 2]. The discovery of the Janus kinase 2 (*JAK2*) V617F mutation significantly improved the understanding of the biology of these disorders [3]. The subsequent identification of other acquired somatic lesions, such as *JAK2 exon 12* mutation [4], mutation in the gene encoding thrombopoietin receptor (*MPL*W*515L/K*) [5] and the recently discovered mutations in the exon 9 of calreticulin (*CALR*) gene [6, 7] reinforced the central role of cytokine receptor/signal transduction lesions in promoting MPN phenotypes. In surveys of large MPN cohorts, *JAK2*V617F can be detected in 95% of patients with PV and *JAK2* exon12 mutation in the remaining 5% of patients. Among ET patients, *JAK2*V617F can be detected in 60 to 65% of patients, *MPL*W515L/K in about 5% and *CALR* mutation in about 20 to 25%. The understanding that an array of somatic mutations contributes to the biology of these disorders [8, 9] prompted new studies to address the impact of patients’ mutation background on disease phenotype. These studies demonstrated that somatic mutations, by determining a more pronounced activation of platelets, leukocytes and endothelial cells [10, 11] and promoting an increased number of leukocytes [12] can sustain a condition of chronic inflammation, which may contribute not only to the premature atherosclerosis underlying the cardiovascular events but also to clonal evolution and second cancer [2, 13–17]. Based on these data, there is an increasing interest in two inflammatory biomarkers, belonging to the superfamily of pentraxins, such as pentraxin 3 (PTX3) and high-sensitivity C-reactive protein (hs-CRP). We hypothesized that these inflammatory markers might be useful to improve prognostic classification of patients with ET and PV. In a previous paper, we showed that PTX3 and hs-CRP levels were correlated with *JAK2* mutation allelic burden and associated with different risks of thrombosis, although in opposite directions [18]. Furthermore, elevated hs-CRP levels were associated with shortened leukaemia-free survival (LFS) in myelofibrosis [19]. However, whether or not somatic mutations, other than *JAK2*V617F, influence the blood levels of PTX3 and hs-CRP is unknown and their association with the main incident relevant outcomes in ET and PV patients has not been fully explored. Accordingly, in this cross-sectional study, we examined a large cohort of patients with PV and ET.

## Methods

Blood samples of 477 patients with ET and PV, diagnosed according to the 2016 World Health Organization (WHO) criteria [20], were obtained from consecutive, well-characterized patients, regularly followed in two Italian haematological centers (Bergamo and Florence). Institutional review board approval was obtained from the two participating centres in the framework of the AIRC-Gruppo Italiano Malattie Mieloproliferative (AGIMM) project. Blood samples were obtained at diagnosis in 172 patients (36%) or during follow-up in 306 (64%) (median time from diagnosis was 4.2 years, range 0.03–29.8 years). The following previously published methods were used: real-time quantitative PCR for *JAK2*V617F [21] and high-resolution melting analysis followed by bidirectional Sanger sequencing or next-generation sequencing for *MPL*W515L/K and *CALR* mutations [22]. In particular, evaluation of *JAK2*V617F mutation was performed on the genomic DNA purified from granulocytes by qualitative or quantitative method. Qualitative assay was based on a allele-specific PCR starting from 100 ng DNA with a sensitivity defined as 0.5–2% [23]. Quantitative assay for the measurement of *JAK2*V617F allele burden was performed by a quantitative real-time PCR assay, using 40 ng DNA. All samples were analysed in triplicate, and the amount of JAK2V617F allele was calculated by comparison with serial dilutions of *JAK2* plasmids. The sensitivity of the quantitative method is 0.08–0.008% [21]. *JAK2*V617F mutation was classified as having low allele burden (<50%, heterozygous) or high allele burden (≥50%, homozygous). High-sensitivity C-reactive protein (hs-CRP) was measured by a latex immunoassay (CardioPhaseHigh Sensitivity Siemens Healthcare Diagnostic Inc., Italy) and PTX3 plasma levels were measured by an in-house Sandwich ELISA as previously described [18].

Major outcomes recorded anytime during the follow-up period were major thrombosis, bleeding, evolution to MF or AL and death. Patients were treated according to current recommendations [24] and therapy included phlebotomy, hydroxyurea, pipobroman, busulfan and alpha interferon. Evolution to post-ET and post-PV MF and AL was diagnosed following the International Working Group for Myeloproliferative Neoplasms Research and Treatment and WHO criteria, respectively [20, 25].

### Statistical analysis

Continuous values were expressed as medians and ranges, and nonparametric K-sample test on the equality of medians was used to test the null hypothesis that the K samples were drawn from populations with the same median. Categorical data were given as counts and percentages; chi-square or Fisher exact test was used to test independence between groups, as appropriate. Boxplots were used to graph distributions across mutational groups. Statistical difference in means of hs-CRP and PTX3 values among mutations was tested considering *JAK2*V617F homozygous category as reference. Bonferroni correction was used to adjust the probability (*p*) values because of the increased risk of the type I error when making multiple statistical tests. Incidence rate of outcomes of interest (major thrombosis, bleeding, haematological evolution and death) were expressed as % patients/year. Logistic regression models were applied to estimate risk prediction of inflammatory biomarkers on outcomes. Multivariable models were evaluated unadjusted and sequentially adjusted for potential confounding factors. All tests for statistical significance were two-tailed and a value of *p* < 0.05 was chosen as the cut-off level for statistical significance. The statistical package STATA for Windows version 12 was used for analysis.

## Results

### General characteristics of the whole population

The 477 patients included 305 with ET (median age 58 years; 63% females) and 172 with PV (median age 58 years; 47% females). Presenting features of the study population are shown in Table 1. Among 305 ET patients, *JAK2*V617F, *MPLW515L/K* and *CALR* mutations were detected in 190 (63%), 14 (5%) and 44 (14%), respectively. The remaining 57 patients (18%) were wild type for all three mutations (triple negative). The group of PV included 95 (55%) patients with heterozygous *JAK2*V617F mutation, 64 (37%) with homozygous *JAK2*V617F mutation, 4 (2%) with *JAK2* exon 12 mutation and 9 (5%) with non-mutated *JAK2*. Median follow-up from diagnosis was 5.4 (range 0.01–30.7) and 6.4 (0.01–30) years in ET and PV, respectively. Aspirin was prescribed in the majority of cases and cytoreductive treatments were given in 57% and 64% of ET and PV cases, respectively. The 306 patients for whom blood samples were obtained during follow-up were receiving antiplatelet agents in the 77 and 60% and cytoreductive treatments in 65 and 68% of ET and PV cases, respectively. The majority of cytoreductive treatments used in this group of patients was hydroxyurea. In all cohorts, none of the patients was in treatment with glucocorticoids at the moment of blood sample collection, while only a minority was receiving statins as usual therapy.

**Table 1:** Patients’ characteristics

	ET	PV	Total
*N*, (%)	305 (64)	172 (36)	477
Sex (M/F), *n* (%)	112/193 (37/63)	91/81 (53/47)	203/274 (43/57)
Age, years, median (5th–95th percentiles)	58.0 (27.7–83.0)	58.3 (35.5–82.0)	58.0 (28.3–82.7)
Mutational status, *n* (%)			
Triple negatives	57 (18)	9 (5)	66 (14)
CALR	44 (14)	0 (0)	44 (9)
MPLW515L/K	14 (5)	0 (0)	14 (3)
JAK2V617F hetero	176 (58)	95 (55)	271 (57)
JAK2V617F homo	14 (5)	64 (37)	78 (16)
JAK2 exon 12 mutation	0 (0)	4 (2)	4 (1)
Hs-CRP, mg/L, median (5th–95th percentiles)	0.83 (0.03–7.96)	0.87 (0.03–6.43)	0.83 (0.03–7.53)
PTX3, ng/mL, median (5th–95th percentiles)	4.55 (0.58–14.95)	5.88 (0.42–22.96)	4.83 (0.54–18.48)

*ET* essential thrombocythemia, *PV* polycythemia vera, *hetero* heterozygous, *homo* homozygous

### Driver mutations and PTX3 and hs-CRP values distribution

**Fig. 1:**
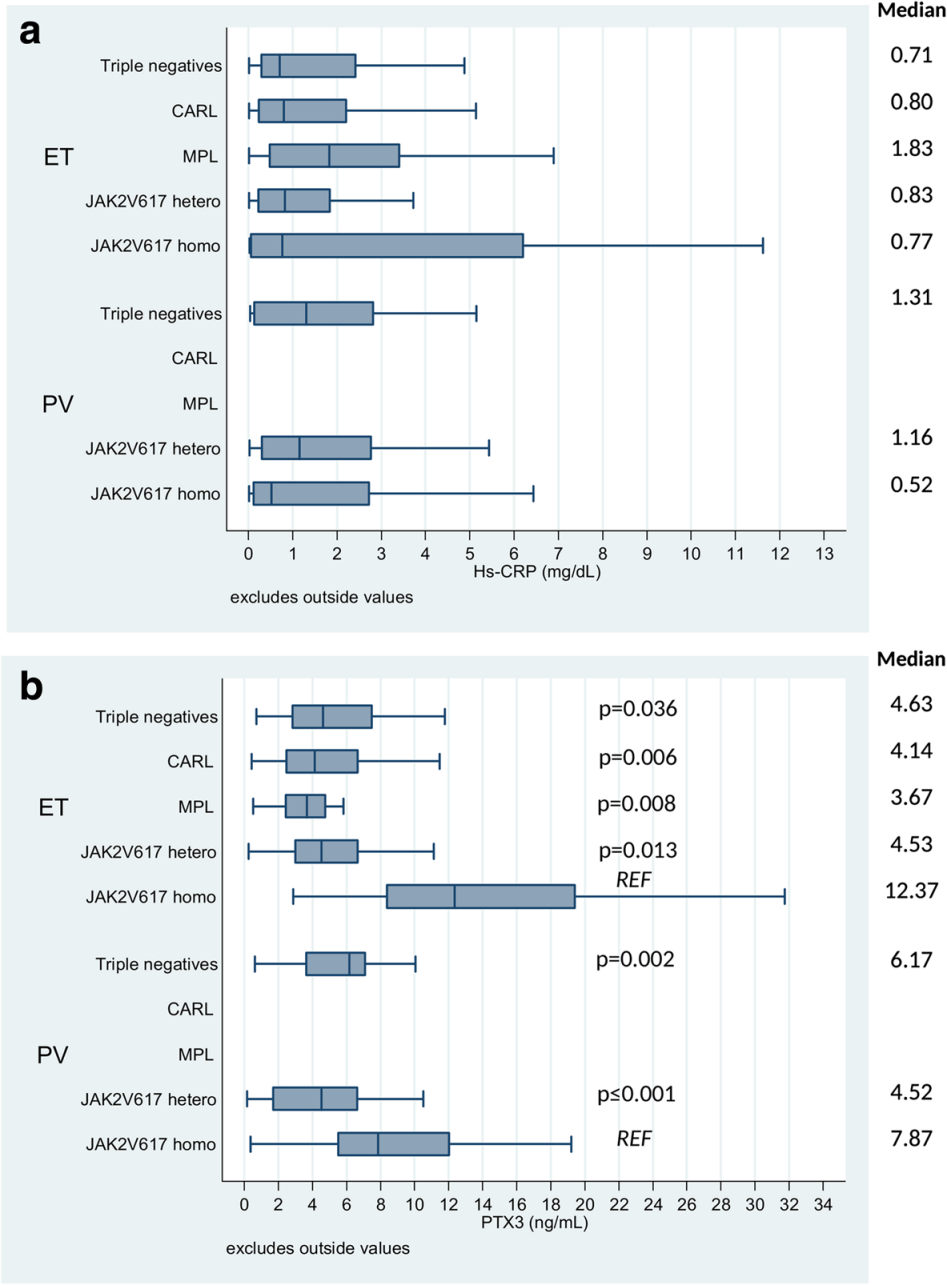
Hs-CRP (**a**) and PTX-3 (**b**) values distribution according to disease and mutational status. Boxplot graphs. Analysis of multiple contrasts versus JAK2V617F homozygous as reference category (Bonferroni adjustment). Only *p* value less than 0.05 are quoted

The median circulating plasma levels of PTX3 detected in ET and PV were similar, being respectively 4.55 ng/mL, range 0.58–14.95 and 5.88 ng/mL, range 0.42–23. Similarly, the median circulating plasma levels of hs-CRP were 0.83 mg/L, range 0.03–7.96 in ET and 0.87 mg/L, range 0.03–6.43 in PV (Table 1). There was no correlation between hs-CRP and PTX3 both in PV and ET (*r* = −0.089, *p* = 0.29 and *r* = 0.027, *p* = 0.64, respectively). In ET patients, hs-CRP levels were similar in triple negative and carriers of *CALR*, *MPL*W*515L/K*, heterozygous *JAK2*V617F or homozygous *JAK2*V617F mutations. In contrast, the circulating levels of PTX3 were significantly increased in homozygous *JAK2*V617F mutation carriers compared to all the other genotypes (Fig. 1). Interestingly, also for PV patients, we observed significantly greater PTX3 blood levels in homozygous *JAK2*V617F mutation carriers compared to heterozygous, while hs-CRP levels were not different by mutational status (Fig. 1). To exclude that the association between PTX3 and *JAK2*V617F homozygous mutation could be affected by other risk factors, we tested whether this relationship remained statistically significant after adjustment for diagnosis (PV vs. ET), gender, age and mutations (*JAK2*V617F homozygous vs. other mutations) in a multivariate model. This analysis confirmed a significant association only between *JAK2*V617F homozygous status and high levels of PTX-3 only (odds ratio (OR) 1.93, 95% confidence interval (CI) 1.44–2.59, *p* < 0.0001).

### Clinical outcomes according to mutational status and hs-CRP and PTX3 values

Fifty-five major thrombotic events occurring during follow-up were registered. Thirty major thrombotic events were registered before blood sampling. The rate of thrombosis was 2.44% patients/year (95% CI 1.35–4.41). Hemorrhagic events were observed during follow-up in a total of 43 patients (9%); the total rate of bleeding was of 1.25 patients/year (95% CI 0.93–1.69). A total of 33 cases of transformation to MF or AL were documented (28 to MF and 5 to AL), accounting for an estimated incidence of haematological transformation of 0.94% patients/year (95% CI 0.66–1.33). The rate of transformation was 0.57% patients/year (95% CI 0.32–1.00) and 1.56% patients/year (95% CI 1.01–2.41) in ET and PV patients, respectively. Among patients taking cytoreductive therapy, the incidence of haematological transformation was 1.19% patients/year (95% CI 0.83–1.71) as opposed to 0.31% (95% CI 0.10–0.93) of patients without cytoreductive treatment. The percentage of transformation among 57 triple negative and 248 mutated ET was 0.36% patients/year (95% CI 0.05–2.54) and 0.99% patients/year (95% CI 0.69–1.41), respectively.

During the time of this study, there were 56 deaths accounting for a mortality rate of 1.58% patients/year (95% CI 1.22–2.06).

To test the hypothesis that different levels of hs-CRP and PTX3 are associated with different outcomes, we categorized PTX3 and hs-CRP using their respective 50th percentile of distribution in the whole cohort as cut-off point and, accordingly, we divided the study cohort in high level group for values above the 50th percentile and reference group for values below the 50th percentile. Clinical outcomes associated with high levels of PTX3 and hs-CRP are presented in Table 2. The crude relative risk for haematological evolution and death associated with PTX3 levels above the 50th percentile (≥4.8 ng/ml) were 1.84 (95% CI, 1.02–3.96) and 1.58 (95% CI, 1.10–2.84), respectively. The multivariable analysis, after sequential adjustment for diagnosis (PV vs. ET), gender, age, treatments (cytoreductive treatments and antiplatelet agents) and mutations showed that the main factor accounting for the excess of haematological evolution and deaths was the presence of *JAK2*V617F homozygous status, as well as the need of treatments (Table 2). In contrast, high levels of PTX3 were associated with a trend to a lower risk of thrombosis also after adjustment for *JAK2*V617F mutation (OR 0.57, 95% CI 0.31–1.04), suggesting a possible protective effect of PTX3. In patients with high hs-CRP levels (>50th percentile, ≥0.8 mg/L) the risk of haematological evolution and death were significantly increased compared to reference group (OR 2.70, 95% CI 1.23–5.95 and OR 3.93, 95% CI 2.01–7.68, respectively). Moreover, patients in the high hs-CRP group suffered also from an excess of thrombosis (OR 2.57, 95% CI 1.39–4.75). It is worth noting that hs-CRP proved an independent risk factor for adverse events in sequential multivariate analysis, also after adjustment for *JAK2*V617F high allele burden (Table 2). Patients in the high PTX3 or hs-CRP group showed a similar risk of bleeding compared to reference groups (OR 1.07, 95% CI 0.57–2.02 and OR 0.77, 95% CI 0.41–1.45, respectively) (Table 2).

**Table 2:** Unadjusted and sequentially multivariable adjusted risk of principal outcomes in ET and PV patients associated to hs-CRP and PTX-3 values over their respective medians

	Hs-CRP ≥0.8 mg/L^a^			PTX-3 ≥4.8 ng/mL^a^		
	OR	95% CI	*p*	OR	95% CI	*p*
Thrombosis (*n* = 55)						
Unadjusted	*2.57*	*1.39–4.75*	*0.003*	0.66	0.37–1.16	0.145
Adjusted for						
+ Male sex	*2.57*	*1.39–4.75*	*0.003*	0.66	0.37–1.16	0.146
+ Age at diagnosis	*2.56*	*1.38–4.73*	*0.003*	0.65	0.37–1.15	0.139
+ PV disease	*2.59*	*1.40–4.81*	*0.003*	0.60	0.34–1.07	0.085
+ Cytoreduction + antiplatelet agents	*2.60*	*1.37–4.95*	*0.003*	0.57	0.31–1.04	0.068
+ JAK2V617F homo	*2.60*	*1.37–4.95*	*0.003*	0.57	0.31–1.04	0.066
Bleeding (*n* = 43)						
Unadjusted	0.77	0.41–1.45	0.419	1.07	0.57–2.02	0.831
Adjusted for						
+ Male sex	0.77	0.41–1.46	0.428	1.07	0.57–2.01	0.842
+ Age at diagnosis	0.84	0.44–1.61	0.596	1.06	0.56–2.01	0.864
+ PV disease	0.84	0.44–1.62	0.603	0.97	0.50–1.86	0.920
+ Cytoreduction+ antiplatelet agents	0.82	0.43–1.59	0.557	0.95	0.49–1.84	0.883
+ JAK2V617F homo	0.83	0.43–1.60	0.574	0.92	0.48–1.79	0.815
Haematological evolution (*n* = 33)						
Unadjusted	*2.70*	*1.23–5.95*	*0.013*	*1.84*	*1.02–3.96*	*0.044*
Adjusted for						
+ Male sex	*2.66*	*1.20–5.86*	*0.015*	*1.87*	*1.02–3.99*	*0.042*
+ Age at diagnosis	*2.61*	*1.18–2.88*	*0.018*	*1.84*	*1.01–3.98*	*0.046*
+ PV disease	*2.70*	*1.21–6.01*	*0.015*	1.59	0.72–3.48	0.251
+ Cytoreduction + antiplatelet agents	*2.72*	*1.21–6.15*	*0.016*	1.64	0.74–3.63	0.225
+ JAK2V617F homo	*2.79*	*1.23–6.32*	*0.014*	1.55	0.69–3.46	0.286
Death (*n* = 56)						
Unadjusted	*3.93*	*2.01–7.68*	*<.0001*	*1.58*	*1.10–2.84*	*0.039*
Adjusted for						
+ Male sex	*3.86*	*1.98–7.55*	*<.0001*	*1.61*	*1.09–2.85*	*0.041*
+ Age at diagnosis	*4.45*	*2.16–9.15*	*<.0001*	*1.56*	*1.05–2.44*	*0.042*
+ PV disease	*4.65*	*2.24–9.65*	*<.0001*	1.14	0.60–2.17	0.680
+ Cytoreduction + antiplatelet agents	*5.26*	*2.47–11.2*	*<.0001*	1.16	0.60–2.22	0.662
+ JAK2V617F homo	*5.41*	*2.53–11.6*	*<.0001*	1.11	0.58–2.15	0.750

*OR* odds ratio, *CI* confidence interval, *p p* value, *PV* polycythemia vera, *ASA* acetylsalicylic acid

^a^Reference categories: hs-CRP <0.8 mg/L; PTX3 <4.8 ng/mL

The variables resulted statistically significant are reported in italicized style

## Discussion

This paper conducted on a large population of ET and PV patients shows a strong association between the levels of PTX3 and *JAK2*V617F allele burden and also indicate that PTX3 and hs-CRP both reflect a condition of chronic inflammation which is associated with the disease severity.

The strong association between the levels of PTX3 and the *JAK2*V617F allele burden is consistent with our previous results [18] and considering the major role played by *JAK2*V617F mutation in the pathogenesis of inflammatory state in MPNs [10] is not surprising. Indeed, it is well established that *JAK2*V617F determines a more pronounced activation of platelets, leucocytes and endothelial cells [10–12] and it might induce the accumulation of reactive oxygen species in the haematopoietic stem cell compartment [26], orchestrating the construction of the inflammatory microenvironment of MPNs [27]. The association of high PTX3 levels and *JAK2*V617F homozygous status with haematological evolution and all-cause mortality suggests that inflammation in the tumour microenvironment might contribute to the genetic instability and disease progression of MPNs [15, 28]. Although it could be argued that only hematological transformation in AML may be due to additional mutation acquisition, the relative small number of total events prevented a formal evaluation of differences by keeping a clear distinction between the MF and AML evolution. In multivariate analysis, after adjustment for *JAK2V617* allele burden, the association between PTX3 levels and adverse outcomes was no longer statistically significant, suggesting that PTX3 levels might simply reflect inflammation derived from constitutively activated blood cells, belonging to the malignant clone harbouring *JAK2*V617F mutation. A second relevant finding, in keeping with our previous results [18], was that the major thrombosis rate tended to be lower at the highest PTX3 levels, suggesting that PTX3 might have a protective role against the detrimental effects of inflammation in the cardiovascular risk. Preclinical studies have shown that PTX3 is released from activated leukocytes and attenuates neutrophil recruitment at sites of inflammation [29], by limiting P-selectin-dependent inflammation [30]. Moreover, PTX3 deficiency was associated with increased fibrin deposition in different models of tissue damage and a direct fibrinolytic effect of PTX3 has been described in vitro [31]*.* In addition, PTX3 administration resulted to be protective in a model of arterial thrombosis [32], and in carcinogenesis models, it has been demonstrated that PTX3 acts as extrinsic oncosupressor by regulating complement-mediated and macrophage-sustained tumour inflammation [33]. While it is possible that *JAK2*V617F mutation is a potent driver of PTX3 release, the biologic and clinical correlates that can be attributed to high levels of circulating PTX3 remain to be defined and interpretations are mostly speculative. In this regard, that PTX3 might play a role in disease progression and development of fibrosis is a distinct possibility [34, 35].

In contrast to PTX3, hs-CRP levels were not associated with the *JAK2*V617F allele burden, supporting the hypothesis that hs-CRP and PTX3 reflect distinct aspects of the inflammatory process and that different mechanisms regulate the circulating levels of these proteins. Patients with a more pronounced chronic inflammation, as suggested by the high hs-CRP levels, exhibited an increased risk of haematological evolution and death of approximately three- and four fold, respectively, and to a lower extent also of thrombotic events compared to those in the lowest group. These findings are in keeping with previous studies in apparently healthy subjects, patients with cardiovascular disease and MPNs patients overall showing that higher levels hs-CRP are associated with an increased risk of all-cause mortality, malignant diseases and thrombotic events [18, 19, 35–39]. A striking finding of our results is that the magnitude of predictive capacity of adverse outcomes of hs-CRP seems to be significantly greater in ET and PV patients than in subjects with and without cardiovascular disease in whom the risk was only about 1.5-fold increased in individuals with highest hs-CRP levels compared to those with lowest levels [37, 40, 41].

We acknowledge that our study has some limitations. First, PTX3 and hs-CRP were measured only once and we could not account for intra-individual variations. In this regard, future studies with prospective, repeated evaluations of the two biomarkers will be necessary to validate their predictive value for prognostic classification. Second, biomarker measurements were not performed at the time of diagnosis in many patients; thus, we cannot ascertain whether elevated PTX-3 and hs-CRP may represent sensitive markers of poor prognosis since the diagnosis, or rather along with disease duration. However, this limitation does not negatively affect the validity of established correlation between high levels of PTX3 and *JAK2*V617F homozygous status, as well as the prognostic relevance of both biomarkers, which reflect the main hypotheses of the current study. Finally, the mechanisms by which PTX3 and hs-CRP are associated with adverse outcomes have not been addressed and our data do not allow to distinguish between the equally tenable explanations that the two biomarkers are simply a marker or rather are mechanistic determinants of patients’ total inflammatory burden. On the other hand, the strength of this study is the high number of well-characterized and molecularly annotated patients that were evaluated with incident assessed outcomes.

## Conclusions

The strong correlation between *JAK2*V617F allele burden and PTX3 levels provides further evidence to support the role of the *JAK2*V617F mutation as a key driver of the MPN-associated chronic inflammation. These results may also represent the groundwork to perform prospective studies aimed at evaluating the impact of treatments targeting simultaneously the clonal haematopoiesis (for example, with interferon-alpha2) and the accompanying inflammatory status (with agents such as JAK1/2 inhibitors, statins and histone deacetylase inhibitors) on the risk of inflammation-driven disease progression and cardiovascular events, especially in patients with high PTX3 and hs-CRP levels.

## Abbreviations

AGIMM: AIRC-Gruppo Italiano Malattie Mieloproliferative
AL: Acute leukaemia
*CALR*: Calreticulin
CI: Confidence interval
ET: Essential thrombocythemia
hs-CRP: High-sensitivity C-reactive protein
LFS: Leukaemia-free survival
MF: Myelofibrosis
MPNs: Myeloproliferative neoplasms
OR: Odds ratio
PTX3: Pentraxin 3
PV: Polycythemia vera
WHO: World Health Organization
